# Barnum’s Broke

**Published:** 1856-04

**Authors:** 


### "BARNUM'S BROKE,"

Is the expressive alliteration on the street, and passes from mouth to mouth with wonderful volubility, with glee, with sorrow, with spiteful delight, according to the heart of the utterer. "Republics are ungrateful," is not more a truth than that the one-eyed "Public," is an unjust judge. The religious newspapers incline to the idea, that Barnum's failure is a Providential dispensation, meaning thereby, that the Almighty has compassed this failure as a merited punishment for the alleged misdeeds of this world-renowned man. We do not take that view of the subject. Our Creator has framed certain laws, physical, mental, moral, for the government of us, his creatures; laws whose observance brings happiness, whose infraction brings suffering, sorrow, degradation, and death itself. To these laws all are amenable, whether pauper or potentate, whether civilized or savage, whether philosopher or fool. Some err on the side of goodness and humanity, others on the side of a wicked nature. A man who has so much of the milk of human kindness in him, as never to be able to refuse a friend a dollar or an endorsement, inevitably comes out at the little end of the horn, is beggared. The miser often clutches so closely, as to lose all. It seems to us, that Barnum's failure is the direct result of kindly influences, and the curse justly falls, and will fall, sooner or later, in terrible dishonor, which is worse than death, on the heart that abused that kindness, that imposed on that confidence.

We have no personal acquaintance with Mr. Barnum, we know him on the street, that is all, and what we say is for a public lesson of some considerable practical importance. In our estimation, and in the estimation of business men of reflection, Phineas T. Barnum was never more of a man than he is at this moment. As to what he has been, we interpret his past life differently from some people. What he is now, what he has been of recent years is the question which ought to determine his *status* among us. No man knows that he is not a knave, until he is "broke," until he has failed in business. It is comparatively easy to be honest when surrounded with abundance, when there are no real, strong temptations to be otherwise. But when the day of failure is foreshadowed unmistakably, and a man finds himself surrounded with bags of gold, which will buy anything, and knows that to-morrow every one of them will be swept away from his grasp, and not a copper be left to buy him a cake to dine upon, under such circumstances, to stand up and be a man, and say to creditors, "*here it is, take it all!*"—this is the ordeal which tries the stuff a man is is made of. That ordeal Barnum has passed safe, unscathed, gloriously for himself, for he has shown himself to be in every inch, a MAN. On his trial, as reported in the *New-York Daily Times*, there was exhibited a promptness, a frankness, a fulness of reply to every question put, with a design to ferret out any concealed property, that from its very uncommonness was amazing, and was as honorable as it was uncommon. Not satisfied with this, but exceeding the requirements of the judge, he offered to give up the watch in his pocket, and the diamond on his finger.

How many a man, do we all know, lives in the splendid mansion of this magnificent city, surrounded with every comfort which money can procure, out of whom innumerable creditors cannot force a farthing, and yet, who are treated with consideration, and spoken of in terms of respect, although continuing as they do, to live in idle ease. Has Barnum done this? Why no, he is too honestly independent, he has gone to keeping a Boarding House, dependent for the very meat and potatoes which are placed upon his table, not on the money covertly laid aside for a rainy day, but upon the confidence of the few friends and kindred who know, better than the *Public*, what the "*great showman*" really is.

How is it, why many a poor milk and water fellow would have gone instanter and blown his brains out, or fed himself to the fishes of the Hudson, and by this time would have been partitioned out to the stomach of the beauty and the beast of great Gotham, this being "Lent." But Barnum was wiser than that, having made a living by feeding the curiosity of New-Yorkers, he had no idea of going to the death, and filling up their paunches with himself. In short, Mr. Barnum has been honorable enough to make himself poor, has been brave enough to go to work for a living, poor enough to labor, but too independent to beg or lean on others for a support, and a grand thing it would be for the *morale* of this community, if thousands among us would "*go and do likewise!*

To make this article applicable to a Journal like ours, we have only to add, it is doleful business to be sick and poor too, and the best possible way of getting rid of two such undesirable attendants, is to go to work like Barnum, and participate in his hearty, rugged, robust health. But as to the money, if you don't be spry, he will get his share first; for it is just as impossible for a man of such ceaseless activity and unconquerable energy to remain poor, as for an Editor to write a "first-rate" notice without being paid for it, or for a Doctor to prescribe efficiently, without a fee.

